# Supplementary material for: A gene horizontally transferred from bacteria protects arthropods from host plant cyanide poisoning
Source: eLife. 2014 Apr 24;3:e02365. doi: 10.7554/eLife.02365 (PMC4011162; doi:10.7554/eLife.02365)
Supplement: Supplementary file 1. — Differentially expressed genes in the London-CYANO strain compared to the London strain. DOI: http://dx.doi.org/10.7554/eLife.02365.013 [file elife02365s001.docx]

Table S1. **Differentially expressed genes in the London-CYANO strain compared to the London strain.**

| Tetur ID | Fold Change | FDR corrected  p-value | Tetur description |
| --- | --- | --- | --- |
| tetur31g01870 | 36.44 | 0.04358 | Hypothetical protein |
| tetur31g00830 | 23.69 | 0.04358 | Hypothetical protein |
| tetur31g00810 | 21.52 | 0.04407 | Hypothetical protein |
| tetur31g00690 | 20.28 | 0.04358 | Hypothetical protein |
| tetur01g09760 | 13.66 | 0.04358 | Hypothetical protein |
| tetur06g02580 | 6.94 | 0.04962 | Cysteine peptidase (C1A papain) |
| tetur07g06460 | 6.76 | 0.04407 | Cytochrome P450 monooxygenase (CYP392A3) |
| tetur07g06410 | 6.16 | 0.04407 | Cytochrome P450 monooxygenase (CYP392A1) |
| tetur07g06440 | 6.10 | 0.04499 | Cytochrome P450 monooxygenase (CYP392A2p) |
| tetur14g03430 | 4.89 | 0.04879 | Hypothetical protein |
| tetur10g01570 | 2.47 | 0.04962 | β-cyanoalanine synthase |
| tetur08g06030 | 2.37 | 0.04962 | Cysteine peptidase (C13 legumain) |
| tetur01g13280 | 2.35 | 0.04470 | Small Secreted Protein, family A |
| tetur08g05980 | 2.33 | 0.04407 | Cysteine peptidase (C13 legumain) (pseudogene) |
| tetur22g02200 | 2.24 | 0.04962 | PLAT single domain protein |
| tetur32g00960 | 2.18 | 0.04407 | Hypothetical protein |
| tetur01g12890 | 2.03 | 0.04649 | Hypothetical protein |
| tetur01g08910 | 2.03 | 0.04962 | Sulfatase |
| tetur04g09469 | -2.04 | 0.04358 | Small Secreted Protein, Family F |
| tetur04g09459 | -2.04 | 0.04358 | Small Secreted Protein, Family F |
| tetur07g00490 | -2.06 | 0.04358 | Hypothetical protein |
| tetur06g02560 | -2.29 | 0.04477 | Hypothetical protein |
| tetur16g03780 | -3.64 | 0.04407 | Apple-like domain (IPR003609) |
| tetur06g05090 | -4.58 | 0.04407 | Short chain dehydrogenase |
| tetur03g10063 | -7.04 | 0.04407 | Small Secreted Protein, Family B |
| tetur03g08840 | -11.33 | 0.04879 | Type II Keratin-like protein |
| tetur03g08830 | -20.61 | 0.04713 | Chemosensory Receptor |
| tetur12g00590 | -40.98 | 0.04407 | Short chain dehydrogenase |

Absolute FC ≥ 2 and FDR corrected p-value < 0.05 were used as cutoffs to evaluate significant differential expression. Tetur IDs can be retrieved at the ORCAE genome portal: <http://bioinformatics.psb.ugent.be/orcae/overview/Tetur>.
