# Supplementary material for: A gene horizontally transferred from bacteria protects arthropods from host plant cyanide poisoning
Source: eLife. 2014 Apr 24;3:e02365. doi: 10.7554/eLife.02365 (PMC4011162; doi:10.7554/eLife.02365)
Supplement: Supplementary file 2. — Genome portals of arthropods consulted in order to retrieve potential homologues of Tu-CAS in addition to NCBI nr/nt databases. DOI: http://dx.doi.org/10.7554/eLife.02365.014 [file elife02365s002.doc]

**Supplementary File 2. Genome portals of arthropods consulted in order to retrieve potential homologues of Tu-CAS in addition to NCBI nr/nt databases.**

| **Subphylum** | **Order** | **Species** | **Genome portal** | **Hom** | **Cont** |
| --- | --- | --- | --- | --- | --- |
|  |  |  |  |  |  |
| **Hexapoda** |  |  |  |  |  |
|  | **Lepidoptera** |  |  |  |  |
|  |  | *Bombyx mori* | [http://www.silkdb.org/silkdb](http://www.silkdb.org/silkdb/#http://www.silkdb.org/silkdb), <http://silkdb.genomics.org.cn/silkworm/> | Yes |  |
|  |  | *Danaus plexippus* | http://monarchbase.umassmed.edu/ | Yes |  |
|  |  | *Heliconius erato* | http://etylus.bio.uci.edu/ | Yes |  |
|  |  | *Heliconius melpomene* | http://etylus.bio.uci.edu/ | Yes |  |
|  |  | *Manduca sexta* | <http://agripestbase.org/manduca/> | Yes |  |
|  |  | *Plutella xylostella* | http://iae.fafu.edu.cn/DBM/ | Yes |  |
|  |  |  |  |  |  |
|  | **Trichoptera** |  |  |  |  |
|  |  | *Limnephilus lunatus* | http://www.hgsc.bcm.tmc.edu/ | No | * |
|  |  |  |  |  |  |
|  | **Diptera** |  |  |  |  |
|  |  | *Aedes aegypti* | https://www.vectorbase.org/ | No | * |
|  |  | *Ceratitis capitata* | http://www.hgsc.bcm.tmc.edu/ | No | * |
|  |  | *Culex quinquefasciatus* | https://www.vectorbase.org/ | No |  |
|  |  | *Drosophila* sp. | http://flybase.org/ | No |  |
|  |  | *Musca domestica* | https://www.vectorbase.org/ | No |  |
|  |  | *Lutzomyia longipalpis* | https://www.vectorbase.org/ | No |  |
|  |  |  |  |  |  |
|  | **Hymenoptera** |  |  |  |  |
|  |  | *Apis mellifera* | http://hymenopteragenome.org/beebase/ | No |  |
|  |  | *Acromyrmex echinatior* | <http://hymenopteragenome.org/ant_genomes/> | No |  |
|  |  | *Athalia rosae* | <http://www.hgsc.bcm.tmc.edu/> | No | * |
|  |  | *Atta cephalotes* | <http://hymenopteragenome.org/ant_genomes/> | No |  |
|  |  | *Bombus impatiens* | http://hymenopteragenome.org/beebase/ | No |  |
|  |  | *Bombus terrestris* | http://hymenopteragenome.org/beebase/ | No |  |
|  |  | *Camponotus floridanus* | <http://hymenopteragenome.org/ant_genomes/> | No |  |
|  |  | *Copidosoma floridanum* | <http://www.hgsc.bcm.tmc.edu/> | No |  |
|  |  | *Harpegnathos saltator* | <http://hymenopteragenome.org/ant_genomes/> | No |  |
|  |  | *Linepithema humile* | <http://hymenopteragenome.org/ant_genomes/> | No |  |
|  |  | *Nasonia vitripennis* | http://hymenopteragenome.org/nasonia/ | No |  |
|  |  | *Orussus abietinus* | http://www.hgsc.bcm.tmc.edu/ | No |  |
|  |  | *Pogonomyrmex barbatus* | <http://hymenopteragenome.org/ant_genomes/> | No |  |
|  |  | *Solenopsis invicta* | <http://hymenopteragenome.org/ant_genomes/> | No |  |
|  |  | *Trichogramma pretiosum* | http://www.hgsc.bcm.tmc.edu/ | No |  |
|  |  |  |  |  |  |
|  | **Strepsiptera** |  |  |  |  |
|  |  | [*Mengenilla moldrzyki*](http://www.ncbi.nlm.nih.gov/genome/13821?project_id=72521) | http://datadryad.org | No |  |
|  |  |  |  |  |  |
|  | **Coleoptera** |  |  |  |  |
|  |  | *Agrilus planipennis* | http://www.hgsc.bcm.tmc.edu/ | No |  |
|  |  | *Anoplophora glabripennis* | http://www.hgsc.bcm.tmc.edu/ | No |  |
|  |  | *Leptinotarsa decemlineata* | http://www.hgsc.bcm.tmc.edu/ | No |  |
|  |  | *Tribolium castaneum* | http://beetlebase.org/ | No |  |
|  |  |  |  |  |  |
|  | **Hemiptera** |  |  |  |  |
|  |  | *Cimex lectularius* | http://www.hgsc.bcm.tmc.edu/ | No | * |
|  |  | *Homalodisca vitripennis* | http://www.hgsc.bcm.tmc.edu/ | No | * |
|  |  | *Oncopeltus fasciatus* | http://www.hgsc.bcm.tmc.edu/ | No |  |
|  |  | *Pachypsylla venusta* | http://www.hgsc.bcm.tmc.edu/ | No |  |
|  |  | *Rhodnius prolixus* | https://www.vectorbase.org/ | No | * |
|  |  |  |  |  |  |
|  | **Heteroptera** |  |  |  |  |
|  |  | *Gerris buenoi* | http://www.hgsc.bcm.tmc.edu | No |  |
|  |  |  |  |  |  |
|  | **Thysanoptera** |  |  |  |  |
|  |  | *Frankliniella occidentalis* | http://www.hgsc.bcm.tmc.edu/ | No | * |
|  |  |  |  |  |  |
|  | **Phthiraptera** |  |  |  |  |
|  |  | *Pediculus humanus* | https://www.vectorbase.org/ | No |  |
|  |  |  |  |  |  |
|  |  |  |  |  |  |

Continued.

| **Subphylum** | **Order** | **Species** | **Genome portal** | **Hom** | **Cont** |
| --- | --- | --- | --- | --- | --- |
|  |  |  |  |  |  |
| **Hexapoda** |  |  |  |  |  |
|  |  |  |  |  |  |
|  | **Orthoptera** |  |  |  |  |
|  |  | *Locusta migratoria* | [http://www.ncbi.nlm.nih.gov](http://www.ncbi.nlm.nih.gov/) (WGS) | No |  |
|  |  |  |  |  |  |
|  | **Odonata** |  |  |  |  |
|  |  | *Ladona fulva* | http://www.hgsc.bcm.tmc.edu/ | No |  |
|  |  |  |  |  |  |
|  | **Ephemeroptera** |  |  |  |  |
|  |  | *Ephemera danica* | http://www.hgsc.bcm.tmc.edu/ | No | * |
|  |  |  |  |  |  |
|  |  |  |  |  |  |
| **Myriapoda** |  |  |  |  |  |
|  | [**Geophilomorpha**](http://nl.wikipedia.org/w/index.php?title=Geophilomorpha&action=edit&redlink=1) |  |  |  |  |
|  |  | *Strigamia maritima* | http://www.hgsc.bcm.tmc.edu/ | No |  |
|  |  |  |  |  |  |
|  |  |  |  |  |  |
| **Crustaceae** |  |  |  |  |  |
|  | **Cladocera** |  |  |  |  |
|  |  | *Daphnia pulex* | http://wfleabase.org/ | No |  |
|  |  | *Daphnia pulicaria* | http://wfleabase.org/ | No |  |
|  | **Copepoda** |  |  |  |  |
|  |  | *Eurytemora affinis* | http://www.hgsc.bcm.tmc.edu/ | No |  |
|  |  | *Lepeophtheirus salmonis* | <http://web.uvic.ca/grasp/gils/> (WGS) | No | * |
|  |  |  |  |  |  |
|  |  |  |  |  |  |
| **Chelicerata** |  |  |  |  |  |
|  | **Araneae** |  |  |  |  |
|  |  | *Latrodectus hesperus* | <http://www.hgsc.bcm.tmc.edu/> | No |  |
|  |  | *Loxosceles reclusa* | <http://www.hgsc.bcm.tmc.edu/> | No |  |
|  |  | *Parasteatoda tepidariorum* | <http://www.hgsc.bcm.tmc.edu/> | No |  |
|  |  |  |  |  |  |
|  | **Ixodida** |  |  |  |  |
|  |  | *Ixodes scapularis* | https://www.vectorbase.org/ | No |  |
|  |  |  |  |  |  |
|  | **Scorpiones** |  |  |  |  |
|  |  | *Centruroides sculpturatus* | http://www.hgsc.bcm.tmc.edu/ | No |  |
|  |  | *Mesobuthus martensii* | [http://www.ncbi.nlm.nih.gov](http://www.ncbi.nlm.nih.gov/) (WGS) | No | * |
|  |  |  |  |  |  |
|  | **Mesostigmata** |  |  |  |  |
|  |  | *Metaseiulus occidentalis* | http://www.hgsc.bcm.tmc.edu/ | No |  |
|  |  | *Varroa destructor* | [http://www.ncbi.nlm.nih.gov](http://www.ncbi.nlm.nih.gov/) (WGS) | No | * |
|  |  |  |  |  |  |
|  |  |  |  |  |  |
|  |  |  |  |  |  |
|  |  |  |  |  |  |
|  |  |  |  |  |  |

Potential Tu-CAS homologues were identified by a tBLaSTn-search using Tu-CAS as the query up to February 2014. The ‘Hom’ column indicates whether a homologue was found. Retrieved homologues were used in the phylogenetic analysis; the corresponding accession numbers can be found in Supplementary File 3. The ‘Cont’ column shows with an asterisk the presence of bacterial cysteine synthases on contaminating bacterial scaffolds/contigs in the species assembly.
