# Supplementary material for: A gene horizontally transferred from bacteria protects arthropods from host plant cyanide poisoning
Source: eLife. 2014 Apr 24;3:e02365. doi: 10.7554/eLife.02365 (PMC4011162; doi:10.7554/eLife.02365)
Supplement: Supplementary file 3. — List of the pyridoxal-5′-phosphate-dependent protein sequences used in phylogenetic tree construction. DOI: http://dx.doi.org/10.7554/eLife.02365.015 [file elife02365s003.docx]

Supplementary File 3. **List of the pyridoxal-5’-phosphate-dependent protein sequences used in phylogenetic tree construction**.

| **Kingdom** | **Species** | **CDD - motif** | **Trivial name** | **Accession #** | **SL** |
| --- | --- | --- | --- | --- | --- |
| Fungi | *Aspergillus fumigatus* | COG0031 | cysteine synthase (CysK) | XP_748124.1 | M |
|  | *Debaryomyces hansenii* | COG0031 | cysteine synthase (CysK) | XP_461064.1 | M |
|  | [*Podospora anserina*](http://www.uniprot.org/taxonomy/515849) | TIGR01137 | cystathionine β-synthase | CAP68365.1 | C |
|  | *Rhizopus delemar* | COG0031 | cysteine synthase (CysK) | EIE81016.1 | M |
|  | *Schizosaccharomyces pombe* | COG0031 | cysteine synthase (CysK) | NP_595332.1 | M |
|  | [*Sclerotinia sclerotiorum*](http://www.uniprot.org/taxonomy/665079) | TIGR01137 | cystathionine β-synthase | EDN93051.1 | C |
|  | [*Talaromyce stipitatus*](http://www.uniprot.org/taxonomy/441959) | TIGR01137 | cystathionine β-synthase | B8M103 | C |
|  | *Trichoderma reesei* | COG0031 | cysteine synthase (CysK) | EGR50915.1 | M |
| Chromalveolata | *Phytophthora infestans* | PLN02565 | cysteine synthase | XP_002900124.1 | M |
|  | *Phytophthora ramorum* | PLN02565 | cysteine synthase | Phyra1_1 library ^●^ | C |
|  | *Phytophthora sojae* | PLN02565 | cysteine synthase | Physo3 ^●^ | M |
| Arthropoda | *Apis mellifera* | TIGR01137 | cystathionine β-synthase | NP_001035353.1 | C |
|  | *Bicyclus anynana* | COG0031 | cysteine synthase (CysK) | GE682124, GE682125 | C |
|  | *Bombyx mori* | TIGR01137 | cystathionine β-synthase | BGIBMGA000065 ^†^ | IM |
|  |  | COG0031 | cysteine synthase (CysK) | BGIBMGA012123 ^†^ | Ex |
|  | *Culex quinquefasciatus* | TIGR01137 | cystathionine β-synthase | XP_001863044.1 | C |
|  | *Danaus plexippus* | TIGR01137 | cystathionine β-synthase | [DPOGS118427](http://monarchbase.umassmed.edu/tools/Get_gene.cgi?id=DPOGS118427-PA) ^‡^ | C |
|  |  | COG0031 | cysteine synthase (CysK) (1) | DPOGS121959 ^‡^ | M |
|  |  | COG0031 | cysteine synthase (CysK) (2) | DPOGS122308 ^‡^ | M |
|  | *Drosophila melanogaster* | TIGR01137 | cystathionine β-synthase | NP_608424.1 | C |
|  | *Heliconius erato* |  | cysteine synthase (CysK) | IC33431AfEcon9573 ^◊^ | C |
|  | *Heliconius melpomene* | TIGR01137 | cystathionine β-synthase | HMEL014964 ^◊^ | C |
|  |  | COG0031 | cysteine synthase (CysK) (1) | HMEL013489 ^◊^ | ER |
|  |  | COG0031 | cysteine synthase (CysK) (2) | HMEL016300 ^◊^ | C |
|  |  | COG0031 | cysteine synthase (CysK) (3) | HMEL002400 ^◊^ | C |
|  | *Heliothis virescens* | COG0031 | cysteine synthase (CysK) | EY121932.1, GR959864.1 | C |
|  | *Manduca sexta* | COG0031 | cysteine synthase (CysK) | Msex007138 ^▲▲^ | C |
|  |  | TIGR01137 | cystathionine β-synthase | Msex006186 ^▲▲^ | C |
|  | *Metaseiulus occidentalis* | TIGR01137 | cystathionine β-synthase | XP_003739386.1 | C |
|  | *Nasonia vitripennis* | TIGR01137 | cystathionine β-synthase | XP_003424942.1 | C/N |
|  | *Papilio xuthus* | COG0031 | cysteine synthase (CysK) | AK402113.1 | C |
|  | *Panonychus citri* | COG0031 | cysteine synthase (CysK) | ERP000885^▲▲▲^ | C |
|  | *Planococcus citri* | COG0031 | cysteine synthase (CysK) | AGR65705.1 | C |
|  | *Plutella xylostella* | TIGR01137 | cystathionine β-synthase | Px003351 ^▼^ | N |
|  |  | COG0031 | cysteine synthase (CysK) | Px003388 ^▼^ | C |
|  | *Spodoptera exigua* | COG0031 | cysteine synthase (CysK) | [Se1E](http://bioweb.ensam.inra.fr/Spodopterav3/tree?name=Se1E&class=Library) library ^▲^ | C |
|  | *Spodoptera frugiperda* | COG0031 | cysteine synthase (CysK) | FP354875.1, FP359108.1 | C |
|  | *Spodoptera littoralis* | COG0031 | cysteine synthase (CysK) | FQ015723.1 | C |
|  | *Tetranychus evansi* | COG0031 | cysteine synthase (CysK) | KF981736 | C |
|  | *Tetranychus urticae* | TIGR01137 | cystathionine β-synthase | tetur06g06721 * | C |
|  |  | COG0031 | β-cyanoalanine synthase | tetur10g01570 * | C |
|  | *Trichoplusia ni* | COG0031 | Cysteine synthase (CysK) | FF378281.1 | M |
|  | *Zygaena filipendulae* | COG0031 | Cysteine synthase (CysK) | c271** and c2894** | C |
| Mammalia | *Homo sapiens* | TIGR01137 | cystathionine β-synthase | NP_000062.1 | IM |
|  | *Mus musculus* | TIGR01137 | cystathionine β-synthase | NP_659104.1 | C |

| **Kingdom** | **Species** | **CDD-motif** | **Trivial name** | **Accession** | **SL** |
| --- | --- | --- | --- | --- | --- |
| Nematoda | *Bursaphelenchus xylophilus* | COG0031 | cystathionine β-synthase | BUX_s00713.518.1 *** | C |
|  |  | PLN02565 | cysteine synthase (1) | BUX_s01144.311 *** | C |
|  |  | PLN02565 | cysteine synthase (2) | BUX_s01109.131.1 *** | C |
|  |  | COG0031 | cysteine synthase (3) | BUX_s00083.34.1 *** | C |
|  |  | COG0031 | cysteine synthase (4) | BUX_s01063.67.1 *** | C |
|  |  | COG0031 | cysteine synthase (5) | BUX_s00083.35.1 *** | C |
|  | *Caenorhabditis elegans* | COG0031 | cystathionine β-synthase | NP_001257091.1 | C |
|  |  | PLN02565 | cysteine synthase (*cysl* 1) | C17G1.7 ^▐^ | C |
|  |  | COG0031 | β-cyanoalanine synthase (*cysl* 2) | K10H10.2 ^▐^ | C/N |
|  |  | COG0031 | cysteine synthase (*cysl* 3) | R08E5.2 ^▐^ | C |
|  |  | COG0031 | cysteine synthase (*cysl* 4) | F59A7.9 ^▐^ | C |
|  | *Meloidogyne incognita* | COG0031 | cystathionine β-synthase | Minc16543 ^▬^ | C |
|  |  | PLN02565 | cysteine synthase | Minc16056 ^▬^ | C |
| Plantae | *Arabidopsis thaliana* | PLN02565 | cysteine synthase | NP_193224.1 | C/Ch |
|  |  | PLN02565 | β-cyanoalanine synthase | NP_191703.1 | M/Ch |
|  | *Glycine max* | PLN02565 | cysteine synthase | AAL66291.1 | C |
|  |  | PLN02565 | β-cyanoalanine synthase | XP_003534555.1 | M |
|  | *Solanum tuberosum* | PLN02565 | Cysteine synthase | O81154.1 | C |
|  |  | PLN02565 | β-cyanoalanine synthase | Q76MX2.1 | M |
|  | *Zea mays* | PLN02565 | Cysteine synthase | NP_001105469.1 | C |
|  |  | PLN02565 | β-cyanoalanine synthase | ADG60236.1 | M |
| Bacteria | *Achromobacter xylosoxidans* | COG0031 | cysteine synthase (CysK) | EGP46495.1 |  |
|  | *Achromobacter xylosoxidans* | TIGR01138 | cysteine synthase (CysM) | YP_003977776.1 |  |
|  | *Burkholderia pseudomallei* | TIGR01138 | cysteine synthase (CysM) | CAH36513 |  |
|  | *Clostridium difficile* | COG0031 | cysteine synthase (CysK) | YP_001088167.1 |  |
|  | *Desulfosporosinus orientis* | COG0031 | cysteine synthase (CysK) | YP_004970592.1 |  |
|  | *Desulfotomaculum reducens* | COG0031 | cysteine synthase (CysK) | YP_001112720.1 |  |
|  | *Desulfotomaculum ruminis* | COG0031 | cysteine synthase (CysK) | YP_004546168.1 |  |
|  | *Enterobacter* sp. Ag1 | COG0031 | cysteine synthase (CysK) | WP_008459590.1 |  |
|  | *Escherichia coli* | TIGR01138 | cysteine synthase (CysM) | ZP_06649856.1 |  |
|  | *Leptospirillum ferrodiazotrophum* | COG0031 | cysteine synthase (CysK) | EES53648.1 |  |
|  | *Mesorhizobium opportunistum* | COG0031 | cysteine synthase (CysK) | YP_004613037.1 |  |
|  | *Methylobacterium radiotolerans* | COG0031 | cysteine synthase (CysK) | YP_001756672.1 |  |
|  | *Methylobacterium* sp. GXF4 | COG0031 | cysteine synthase (CysK) | ZP_10356649.1 |  |
|  | *Pantoea* sp. At-9b | COG0031 | cysteine synthase (CysK) | YP_004116693.1 |  |
|  | *Photorhabdus luminescens* | TIGR01138 | cysteine synthase (CysM) | NP_928691 |  |
|  | *Pseudomonas putida* | TIGR01138 | cysteine synthase (CysM) | YP_005931751 |  |
|  | *Roseovarius nubinhibens* | COG0031 | cysteine synthase (CysK) | ZP_00959725.1 |  |
|  | *Sagittula stellate* | COG0031 | cysteine synthase (CysK) | ZP_01743962.1 |  |
|  | *Singulisphaera acidiphila* | COG0031 | cysteine synthase (CysK) | ZP_09573263.1 |  |
|  | *Sodalis glossinidius morsitans* | COG0031 | cysteine synthase (CysK) | YP_455380.1 |  |
|  | *Thermosinus carboxydivorans* | COG0031 | cysteine synthase (CysK) | ZP_01666588.1 |  |
|  | *Thermovirga lienii* | COG0031 | cysteine synthase (CysK) | YP_004933490.1 |  |
|  | α-proteobacterium BAL199 | COG0031 | cysteine synthase (CysK) | ZP_02191584.1 |  |
|  | γ-proteobacterium HIMB55 | COG0031 | cysteine synthase (CysK) | ZP_09692532.1 |  |

Continued.

Conserved Domain Database (CDD)-motifs, trivial names, accession numbers and subcellular localization of protein sequences used for phylogenetic analysis. CDD- motifs were identified using the CDD-server (Marchler-Bauer et al., 2011). Subcellular localization (SL) of protein sequences was predicted with WoLF PSORT (Horton et al., 2007): C, cytoplasmic; Ch, chloroplast; ER, endoplasmic reticulum; Ex, extracellular; M, mitochondrial; N, nuclear; IM, integral membrane. Protein sequences were either retrieved from the NCBI/Uniprot database or from individual genome/transcriptome portals. In case of the latter the accession number is followed by a superscript symbol to indicate the genome/transcriptome portal that has been consulted:

^●^ <http://genome.jgi.doe.gov/>; ^†^ [http://www.silkdb.org/silkdb](http://www.silkdb.org/silkdb/#http://www.silkdb.org/silkdb), <http://silkdb.genomics.org.cn/silkworm/> and <http://silkworm.genomics.org.cn/>;^‡^ <http://monarch.umassmed.edu/>; ^◊^ [http://www.butterflygenome.org](http://www.butterflygenome.org/);
^▼^ <http://iae.fafu.edu.cn/DBM/index.php>; ^▲^ <http://bioweb.ensam.inra.fr/spodobase>;^▲▲^ <http://agripestbase.org/manduca/>; ^▲▲▲^ <http://www.ebi.ac.uk/>; * <http://bioinformatics.psb.ugent.be/orcae/overview/Tetur>;**http://genome.ku.dk/resources/zygaena/;*** <http://www.genedb.org/Homepage/Bxylophilus>; ^▐^ <http://www.wormbase.org/#01-23-6>; ^▬^ <http://www.inra.fr/meloidogyne_incognita/>.
