# Supplementary material for: A gene horizontally transferred from bacteria protects arthropods from host plant cyanide poisoning
Source: eLife. 2014 Apr 24;3:e02365. doi: 10.7554/eLife.02365 (PMC4011162; doi:10.7554/eLife.02365)
Supplement: Supplementary file 4. — List of primers used in PCR in T. urticae and T. evansi. DOI: http://dx.doi.org/10.7554/eLife.02365.016 [file elife02365s004.doc]

| **Fragment** | **Species** | **Primer** | **Sequence (5’ - 3’)** | **T_m_ (°C)** |
| --- | --- | --- | --- | --- |
|  |  |  |  |  |
| *tetur10g01550* – *tetur10g01570* |  |  |  |  |
|  | *T. urticae* |  |  |  |
|  |  | **1550_1570_F** | **CCAATAAAGCCATCATCGTG** | **58.99** |
|  |  | **1550_1570_R** | **TGCTTTCCTTGTTGGATGTG** | **59.69** |
|  |  | 1550_1570_seq1 | CGCTAATACGGTCTCCGATG | 60.62 |
|  |  | 1550_1570_seq2 | TTTCAGGCTCAGGAAAATCAG | 59.44 |
|  |  | 1550_1570_seq3 | TGGATGGGGTGGTTATTCC | 60.39 |
|  |  | 1550_1570_seq4 | GCCGGTTTTTACCGTTCTATC | 59.86 |
|  |  | 1550_1570_seq5 | CAGTTCTTATGTACCAGTGATGTCTTG | 60.37 |
|  |  | 1550_1570_seq6 | TTTTCACCTGAGTTTCCAACG | 60.13 |
|  |  | 1550_1570_seq7 | GAGCAAATGAAGAGGCTTGG | 59.96 |
|  | *T. evansi* |  |  |  |
|  |  | **1550_1570_F_eva** | **ATAAAGCCATCATCGTGTTCG** | **59.91** |
|  |  | **1550_1570_R** | **TGCTTTCCTTGTTGGATGTG** | **59.69** |
|  |  | 1550_1570_eva_seq1 | GTGCCCCGTTGAAAATAATG | 60.19 |
|  |  | 1550_1570_eva_seq2 | TCAACCAAACATCCAACCAG | 59.39 |
|  |  | 1550_1570_eva_seq3 | GCGCGCTTCCTTTATTTTC | 59.94 |
|  |  | 1550_1570_eva_seq4 | AAGTTATTCGCCTTCTTGATCC | 58.78 |
|  |  | 1550_1570_eva_seq5 | TCTGTCACTTCCATGTTTACATCC | 60.28 |
|  |  | 1550_1570_eva_seq6 | CAGGATTGTCACTACCCAAGC | 59.61 |
|  |  |  |  |  |
| *tetur10g01570* – *tetur10g01580* |  |  |  |  |
|  | *T. urticae* | **1570_1580_F** | **CCTTGGAGCAAATGAAGAGG** | **59.81** |
|  |  | **1570_1580_R** | **AGCAAACATCAGCAATTTCG** | **58.92** |
|  |  | 1570_1580_seq1 | TGGTTACCGTGAATGGATGAC | 60.63 |
|  |  | 1570_1580_seq2 | AAGAGAGGGAAGTGAAGATACTAGATG | 59.02 |
|  |  | 1570_1580_seq3 | GACGTTTCAGGGATGACCTTC | 60.88 |
|  |  | 1570_1580_seq4 | GAAGGAGCAGAGAGGGAGAAG | 59.71 |
|  |  | 1570_1580_seq5 | CTTCTCGTTAAGGTATGTTTTCCAG | 59.64 |
|  | *T. evansi* |  |  |  |
|  |  | **1570_1580_F** | **CCTTGGAGCAAATGAAGAGG** | **59.81** |
|  |  | **1570_1580_R_eva** | **TTCCAGTCTCTTCCCAAACC** | **59.11** |
|  |  | 1570_1580_eva_seq1 | TCCTTGAGTTTCCCTGCTTC | 59.41 |
|  |  | 1570_1580_eva_seq2 | AATCCCTAACCATTGATTGCTG | 60.20 |
|  |  | 1570_1580_eva_seq3 | CAGCCAAAACATTACAGATTCG | 59.65 |
|  |  | 1570_1580_eva_seq4 | CCGTTTATGCCAGAAGGATG | 60.46 |
|  |  | 1570_1580_eva_seq5 | TATCACCATGCGAAATCAGG | 59.50 |
|  |  |  |  |  |

Supplementary File 4. **List of primers used in PCR in *T. urticae* and *T. evansi.***

Primers in bold were used to amplify the respective genomic fragments in *T. evansi* and/or *T. urticae*. Primer names including ‘seq’ were used for sequencing of the genomic fragments and were designed in *T. evansi* by primer walking.
