# Supplementary material for: A gene horizontally transferred from bacteria protects arthropods from host plant cyanide poisoning
Source: eLife. 2014 Apr 24;3:e02365. doi: 10.7554/eLife.02365 (PMC4011162; doi:10.7554/eLife.02365)
Supplement: Supplementary file 5. — Coding sequences of Tu-CAS. DOI: http://dx.doi.org/10.7554/eLife.02365.017 [file elife02365s005.docx]

Supplementary File 5. **Coding sequences of *Tu-CAS****.*

| Sequence Name | Coding Sequence (5’-3’) |
| --- | --- |
| *Tu-CAS* | ATGACTGAGTCAACTGTCGACCGAATCAATGGTATTACTCCAAGTGCTTTGGATCTCATTGGTAACACTCCGTTAATC GCTCTTGATCGACTATGGCCTGGACCAGGACGACTTTTGGCTAAATGTGAGTTCCTCAATCCTACTGCTAGTCTTAAA GATCGTTCATCATATTACATGATAGCCAAAGCCAAAGAATCAGGCCAACTCAAGGATGGTGAATCAGTGATAGAGGTA ACCTCTGGTAATCAAGGAGGAGGAATAGCTTGTGTAACTGCAGTTATGGGTCATCCATTCACGGTAACCATGTCAAAG GGTAACAGTCCTCAAAGGGCAATAATGATGAATGCTTTGGGAGCCAATGTGATCCTTGTTGATCAAGTAACGGGCAAA CCTGGTAATGTGACTGCTGATGATGTTGCTGCCGCTGAGGAGACCGCAATGAAAATTAGAGAAGAAACCAATGCTTAC TATGTTGATCAATTCAACAATCCTACAAATTGTTTGGCTCATTATGAAACAACAGGACCAGAAATTTGGAGACAAACA AATGGACGAATTGATGCTTTCCTTGTTGGATGTGGAACTGGAGGCTGTTTTGTTGGAACTTCCAAATTTTTAAAAGAA AAGAATCCAAATGTTCGGTGTTTTGTGGTTGAACCAGAGGGTTGCCAACCCATTGCTGGATGCACCATCACCAAGCCT CTTCATTTGCTCCAAGGATCAGGATATGGATGTGTTCCTACTCTTTTTGATAAAAAGGTTTACAATGATTCCATCTCT GTAAGTGACGAAGAAGCAATTGAATATCGAAAGCTTTTAGGCCAAAAAGAGGGACTTTTCTGTGGTTTTACAACAGGT GGTAATATCGCTGCAGCCATAAAACTTTTGAAATCAGGACAGTTACCAAAAGACGCTTGGGTAGTAACGATTCTGTGT GACAGTGGTCTAAAATATCCAGAATAA |
| *Tu-CAS_mod* | ATGACCGAAAGCACCGTGGACCGCATTAACGGCATTACCCCGAGCGCACTGGACCTGATTGGCAACACCCCGCTGATC GCACTGGACCGTCTGTGGCCGGGTCCGGGTCGTCTGCTGGCAAAATGCGAATTTCTGAACCCGACCGCCTCCCTGAAG GACCGTAGCTCTTATTACATGATTGCAAAAGCTAAGGAAAGCGGTCAGCTGAAAGATGGCGAATCTGTCATTGAAGTG ACCAGTGGTAACCAAGGCGGTGGCATCGCATGTGTTACGGCTGTCATGGGTCATCCGTTCACCGTTACGATGTCGAAA GGCAATAGCCCGCAGCGTGCAATTATGATGAACGCGCTGGGCGCCAATGTGATCCTGGTTGATCAAGTCACCGGCAAA CCGGGTAACGTGACGGCTGATGACGTTGCGGCCGCAGAAGAAACCGCAATGAAGATCCGCGAAGAAACGAACGCTTAT TACGTGGACCAGTTTAACAATCCGACCAATTGCCTGGCGCATTATGAAACCACGGGTCCGGAAATTTGGCGTCAAACC AATGGCCGCATCGATGCCTTTCTGGTTGGTTGCGGCACCGGTGGCTGTTTTGTCGGCACGTCGAAATTCCTGAAAGAA AAGAACCCGAATGTTCGTTGCTTCGTGGTTGAACCGGAAGGTTGCCAGCCGATTGCAGGCTGTACCATCACGAAACCG CTGCACCTGCTGCAAGGTAGTGGCTATGGTTGTGTGCCGACCCTGTTCGATAAAAAGGTCTACAACGACAGTATTTCC GTGTCAGATGAAGAAGCCATCGAATACCGCAAACTGCTGGGCCAGAAGGAAGGTCTGTTTTGTGGCTTCACCACGGGT GGCAATATTGCTGCGGCCATCAAACTGCTGAAGTCTGGCCAGCTGCCGAAAGACGCCTGGGTTGTGACCATCCTGTGT GACTCTGGCCTGAAGTATCCGGAATAA |

*Tu-CAS*: coding sequence before codon optimization, *Tu-CAS_mod*: coding sequence after codon optimization. The *Tu-CAS_mod* sequence was used for recombinant expression in *E. coli.*
